# Development and factorial validity of the Psychological Skills Inventory for Sports, Youth Version – Short Form: Assessment of the psychometric properties

**DOI:** 10.1371/journal.pone.0220930

**Published:** 2019-08-15

**Authors:** Boris Milavic, Johnny Padulo, Zoran Grgantov, Mirjana Milić, Stefania Mannarini, Gian Mauro Manzoni, Luca Paolo Ardigò, Alessandro Rossi

**Affiliations:** 1 Faculty of Kinesiology, University of Split, Split, Croatia; 2 Department of Biomedical Sciences for Health, Università degli Studi di Milano, Milan, Italy; 3 Tunisian Research Laboratory Sports Performance Optimization, National Center of Medicine and Science in Sport, Tunis, Tunisia; 4 Interdepartmental Center for Family Research, University of Padova, Padova, Italy; 5 Department of Philosophy, Sociology, Education, and Applied Psychology, Section of Applied Psychology, University of Padova, Padova, Italy; 6 Faculty of Psychology, eCampus University, Novedrate, Italy; 7 Psychology Research Laboratory, Ospedale San Giuseppe, IRCCS, Istituto Auxologico Italiano, Verbania, Italy; 8 School of Exercise and Sport Science, Department of Neurosciences, Biomedicine and Movement Sciences, University of Verona, Verona, Italy; University of Lleida, SPAIN

## Abstract

Researchers in sport often try to investigate relations between athletes’ psychological skills and their sports results to predict top athletic achievements or unexpectedly poor performances. The Psychology Skills Inventory for Sports (Youth version), PSIS-Y, was developed to measure psychological characteristics of young athletes–differentiating well more talented and less talented young athletes. Nevertheless, previous studies revealed its inadequate, factorial validity. Thus, the aim of this study was to develop and investigate the psychometric proprieties of a brief version of the PSIS-Y (PSIS-Y-SF) in a sample of young Croatian athletes. Participants (*n* = 304; 188 females and 116 male) were recruited in clubs/teams all over Croatia and all of them competed in the Croatian Championship in youth (*n* = 157) and *junior* category (*n* = 147). The PSIS-Y-SF was derived by ten expert psychologists with five of them who had past experiences of agonistic sport practice. Psychometric analysis included Confirmatory Factor Analysis (CFA), internal consistency analysis (Raykov’s Maximal Reliability), and correlation between subscales. Moreover, Multivariate Analyses of Variance (MANOVA) was run to test statistical differences between the players’ categories (male youth *vs*. male *junior vs*. female youth *vs*. female *junior*) in all of the subscales. Results of the CFA suggested the adequateness of the supposed six first-order factor solution for the PSIS-Y-SF. The Maximal Reliability statistics suggest a good internal consistency for all of the subscales and the MANOVA suggested differences between the player’s categories. The PSIS-Y-SF resulted to be a valid and reliable tool for the assessment of sports psychological skills. Findings from the psychometric evaluation of PSIS-Y-SF suggest that this is a useful tool, which may further assist in the measurement and conceptualization of sport psychological skills.

## Introduction

According to the Achievement Goal Theory [1,2] and Self-Determination Theory [3], extensive research supported the key role of psychological attributes to promote athletes’ performance [3–5]. Indeed, it has been widely demonstrated how sport success depends on the reciprocal enhancement of an athlete’s physical and technical abilities as well as a functional pattern of psychological sport skills in relation to his/her own sport. This is exactly why researchers try to determine the relations between athletes’ psychological skills and their sports results (most frequently their athletic performance and placement in a sports competition). Morris [6] stated it is not surprising that psychological characteristics often distinguish more successful elite athletes from less successful ones, because all elite athletes are constantly under a high level of pressure.

In the last decades, research has proven that specific psychological factors play a key role in predicting athletes’ success [6–8], some crucial factors are: motivation, self-confidence, mental preparation, emotional management, and mental readiness [7–14] predominantly determined by the measurements at the same time point with athletes’ performance. These factors are characteristic of a competitive and functional mindset that is necessary to reach high performance levels. Several studies [14–16] proved that the mindset really makes the difference, indeed, psychological skills showed to reliably discriminate between more and less successful athletes [6]. Thus, properly assessing the psychological skills involved in sport might be extremely useful both to select the best athletes, both to facilitate the young practitioners to develop mental skills [6] that are fundamental for a high-level sport career. By assessing and identifying the specific potentialities, or issues, of each athlete, is thus possible to strengthen the psychological preparation of athletes, to improve their well-being, and consequently, also their sport results.

A clear delineation of personal sport skills is interesting for several aims, for example to flexibly tailor psychological skills training interventions which can be adapted to different kind of athletes, also those with disabilities [17] in order to enhance their performance in competitions.

Reliable measures of psychological skills are needed also to test the efficacy of evidence-based techniques, such as psychological skills training and/or mindfulness-based interventions [18]. Psychological skills showed their importance in sport across the athletes’ gender and in many different disciplines [10,11,19] a proper assessment represents the first step to conduct valuable studies.

The importance of psychological skills can also be easily noticed in volleyball [20–25], which is a very dynamic team sport characterized by very short rallies, lasting averagely only 7 sec [26], and tight set finals that additionally increase the psychological pressure. Also, constant alteration of very short defensive and attacking activities require every player to constantly use all psychological skills, especially fast attention focusing/refocusing and those skills used by players in coping with unexpected situations during the game, such as self-confidence, peaking under pressure, and control of competitive state anxiety [8].

Mahoney et al. [8] developed a 51-item questionnaire to assess motivation, self-confidence, anxiety control, mental preparation, team orientation, and concentration in 713 male and female athletes from 23 sports from elite to non-elite collegiate level. They constructed a results-driven sport-specific profile for assessing a wide range of psychological skills (Psychological Skills Inventory for Sport questionnaire form [PSIS]) related to top athletic performances. The questionnaire development was based on previous work by Mahoney et al. [8] with athletes at student but also at Olympic level. The original PSIS consisted of 51 items, on a true-or-false response. The revised version of the original PSIS (PSIS-5R) included 45 items assessed by participants on a five-point Likert scale [8]. This version was developed with the aim of distinguishing elite, pre-elite, and student athletes according to their psychological skills. The questionnaire measured six dimensions: mental preparation, motivation, concentration, self-confidence, team emphasis, and anxiety control.

The questionnaire differentiated male and female athletes, athletes of individual sports, and athletes of team sports, as well as groups of athletes at different levels of athletic skill. Statistically significant differences were found between elite athletes and non-elite athletes. Elite athletes were more motivated for achievement in their sport, experienced less problems with anxiety, referenced more internally, prepared themselves more kinesthetically and mentally, were more focused on their performance than their team members, and maintained more their concentration [8]. However, Murphy and Tammern [27], in their review on the psychometric properties of the PSIS-5R questionnaire, stated that the results of the included studies were inconsistent and ambiguous in terms of different aspects of reliability and validity.

In addition, other studies pointed to the inconsistency of the results obtained by comparing athletes of different situational efficacy [7,28,29]. Because of its limitations, Weinberg and Forlenza [30] pointed to the questionable applicability of the PSIS for research and applied purposes.

The Psychology Skills Inventory for Sports–Youth (PSIS-Y) version [31] was one of the first questionnaires, whose multidimensional focus was directed to psychological characteristics of young athletes. It was established that the questionnaire differentiated more talented and less talented young athletes, especially in females, whereas motivation and mental preparation were found to be useful indicators of differences between elite and sub-elite athletes, regardless of the sport type or participants’ gender [32].

However, to our knowledge, only one study investigated the factorial validity of the PSIS-Y. Indeed, only Sindik and colleagues [33] performed a factor analysis (Principal Axis Factoring–PAF) with Promax rotation. Their results revealed an adequate six-factor solution–that explains almost the 55% of variance–with very high internal consistency values (Cronbach’s *alpha*: from 0.829 to 0.916) and the correlations between the six factors did not exceed 0.42. Nevertheless, despite these interesting results, the PAF showed that (A) a very large number of items did not load on the hypothesized factor (*e*.*g*., Factor 1 and Factor 6) and (B) most of the items had high cross loadings values (from 0.404 to 0.552). These results revealed that both the PSIS-5R and the PSIS-Y have not a good, reliable, and stable factorial validity. Moreover, due to their length, both the PSIS-5R and the PSIS-Y are suitable neither for routine evaluation nor for quick and rapid administration [34].

Considering this background, the aim of this study was to develop and investigate the psychometric proprieties of a brief version of the PSIS-Y (PSIS-Y-SF) in a sample of young Croatian volleyball players.

## Materials and methods

### Participants

Sample size calculation was based on the sample size used in the previous study assessing the factorial structure of the PSIS-Y [33] combined with scientific literature guidelines [35–38]. More in detail, on one hand, the study of Sindik et al. [33] involved 172 participants. On the other hand, on the basis of reviews of simulation studies [39], scientific guidelines suggested a minimum sample size of 200 observations for models of moderate complexity [35–38,40–42]. Thus, a sample size of 200 athletes was considered adequate to correctly estimate parameters of a Confirmatory Factor Analysis (CFA [35,38–46]).

All participants were examined in the same season period of the Croatian Championship (CC) tournament. Assessments were completed individually, immediately before the training session. Three hundred and four (*n* = 304) volleyball players (188 females, 61.8%; and 116 males 38.2%) playing in clubs/teams all over Croatia were recruited. Athletes were aged from 14 to 19 yrs (*mean* = 16.28 yrs, *SD* = 1.93 yrs) and all of them competed in CC in youth (157, 51.6%) and/or *junior* category (147, 48.4%). Participants started playing volley from 7 to 17 yrs of age (*mean* = 11.25 yrs, *SD* = 2.30 yrs) and they training from 2 to 28 hours per week (*mean* = 8.98 hrs, *SD* = 3.94 hrs). Finally, 72 (23.7%) athletes performed–at least–one match in the Croatian National Team.

An informed written consent for participating in the research was given by all participants and their parents. The study was approved by the local university ethics committee (University of Split, Human Research Ethics Committee) according to the Declaration of Helsinki.

### Procedures

#### Translation and cross-cultural adaptation of the Psychological Skills Inventory for Sports Youth version–Short Form

According to guidelines [47], the validation procedure started with the translation of the PSIS-Y followed by a consultation with three experts in the field about each item. The Croatian-language PSIS-Y was then administered to a sample of young/*junior* volleyball players (*n* = 30) of both sexes (who did not enter in the research), with the specific purpose of testing the content validity of its items and each item that had been marked as less comprehensive or insufficiently applicable to volleyball was discussed again within the experts’ board.

#### Development of the Psychological Skills Inventory for Sports–Youth version–Short Form

Once obtained the Croatian version of the PSIS-Y, a double-blind study procedure was used to select items of the PSIS-Y to create its short form. An external collaborator submitted PSIS-Y items to ten expert psychologists (five experts in the field–who had past experiences of agonistic sport practice). Both the collaborator and the psychologists were blind about the aim of this procedure and which questionnaire those items were taken from. Experts were asked to indicate–for each factor separately–the three items that most represented its construct: Motivation (MT), Self-Confidence (SC), Anxiety Control (AC), Mental Preparation (MP), Team Emphasis (TE), and Concentration (C). An agreement between experts higher than 80% was considered adequate to retain the items into the PSIS-Y-SF. The 18 items reported in Table 1 constituted the short version of the PSIS-Y: the PSIS-Y-SF.

**Table 1:** Psychological Skills Inventory for Sports–Youth version–Short Form items descriptive statistics.

		M	Medn.	SD	sk.	k	%Min	%Max
***MT*–Motivation**		4.09	4.3	0.800	-0.737	0.016		
1	I am very motivated to do well in my sport	4.21	4	0.917	-1.310	1.890	02.3%	45.7%
2	I want to train hard to belong to the top in my sport	3.81	4	1.117	-0.682	-0.287	03.9%	34.2%
3	I want to succeed in my sport	4.27	5	0.897	-1.207	0.352	00.7%	52.3%
***SC*–Self-confidence**		3.39	3.3	0.789	-0.127	-0.239		
4	In most competitions, I go in confident that I will do well	3.49	3	0.978	-0.187	-0.387	02.3%	16.4%
5	I can usually remain confident even through one of my poorer performances	2.94	3	0.983	0.098	-0.457	05.9%	05.6%
6	I have faith in myself	3.75	4	0.948	-0.494	-0.211	01.3%	22.7%
***AC*–Anxiety Control**		2.71	2.6	0.919	0.214	-0.182		
7	I am often panic-struck during those last few moments before I begin my performance (R)	2.45	2	1.159	0.602	-0.409	22.0%	06.9%
8	Before a meet, I worry if I will do well (R)	3.10	3	1.096	-0.115	-0.420	09.5%	11.2%
9	Before important meets, I feel intense anxiety (R)	2.59	2	1.148	0.419	-0.562	18.1%	07.2%
***MP*–Mental Preparation**		2.78	3	0.981	0.006	-0.629		
10	I often “rehearse” my performance in my head before I perform	2.95	3	1.182	-0.091	-0.877	14.1%	08.9%
11	When I mentally practice my performance, I “see” myself performing–just like I was watching a videotape	2.63	3	1.204	0.252	-0.852	21.7%	07.2%
12	I prepare for a meet by making mental representations of my performance	2.77	3	1.145	0.162	-0.689	15.1%	07.9%
***TE*–Team emphasis**		4.06	4.3	0.756	-0.851	0.550		
13	I think team spirit is very important	4.51	5	0.856	-1.901	3.261	01.0%	69.1%
14	When my team loses, I feel badly–no matter how well I did as an individual	3.94	4	1.081	-0.851	-0.012	03.0%	38.5%
15	If my teammates don’t exert themselves to the utmost, I get angry	3.73	4	1.054	-0.424	-0.550	02.3%	28.6%
***C–*Concentration**		2.28	2.3	0.806	0.485	-0.113		
16	I often have trouble concentrating during my performance (R)	2.45	2	1.052	0.679	-0.047	16.4%	05.6%
17	At the beginning of my performance, I have trouble forgetting things I was doing before (R)	2.28	2	1.074	0.648	-0.209	26.0%	03.6%
18	During my performance, others distract me (R)	2.12	2	0.987	0.846	0.532	29.3%	03.0%

M = mean; Medn. = median; SD = standard deviation; sk. = skewness; k. = kurtosis; %Min = percentage of subjects, who choose the first category of response (1 = strongly disagree); %Max = percentage of subjects who choose the last category of response (5 = strongly agree); (R) = Item reverse.

#### Final version of the Psychological Skills Inventory for Sports–Youth version–Short Form

Thus, the PSIS-Y-SF directly derived from the PSIS-Y [31] that in turn was an adaptation for youth of the PSIS-5R [8]. Whereas the latter consisted of 45 items that were assessed on a five-point Likert scale, the former consisted of 44 items. Both versions measure six dimensions of psychological skills: mental preparation, motivation, concentration, self-confidence, team emphasis, and anxiety control. The PSIS-Y was more adapted to athletes of younger age categories in terms of context as opposed to the original version of the PSIS or the PSIS-5R.

Consequently, the PSIS-Y-SF was an 18 items instrument measuring the same six factors of its previous versions retaining three items per factor. Items were scaled on five-point Likert scale ranging from 1 (= almost never) to 5 (= almost always). The total score of each scale was derived by computing the mean of items of the factor. Thus, it was possible to compute six different scores (one for each scale). According to previous versions of the questionnaire, no PSIS-Y-SF total score could be computed.

### Statistical analysis

Statistical analyses were performed with R software (v 3.4.4 [48]) and using “Lavaan” (v. 0.5–23.1097 [49]) and semPlot (v.1.1.1 [50]) packages. Psychometric analysis included CFA, with item discriminant power (IDP) analysis, internal consistency analysis (Raykov’s Maximal Reliability [MR]). For the present study, critical *P*-value was set to 0.050.

Considering the nature of the response scale [51], the *diagonal weighted least square* (DWLS) estimator was used–as a robust method to non-normality–to assess the factorial structure of the PSIS-Y-SF [35,40,51–53]. Model fit was assessed by using the Chi-square statistics (χ^2^), the Root-Mean Square Error of Approximation (RMSEA), the Comparative Fit Index (CFI), and the Weighted Root Mean Residual (WRMR) [35,40,52–54]. The following cut-off criteria were chosen to evaluate the goodness of fit: statistically non-significance of the χ^2^, an RMSEA lower than 0.08, a CFI higher than 0.95, and a WRMR lower than 1.00 [35,40,52–54].

Moreover, model comparisons were performed to exclude that the PSIS-Y-SF provided a configuration structure other than its long form PSIS-Y [55]: (A) a single factor model, (B) a second order model (hierarchical), and (C) a bi-factor model (hierarchical). According to scientific literature [35,53], the second order model was specified by posit an overarching general latent dimension–called “sport skills”–loaded by the six first-order latent dimensions (MT, SC, AC, MP, TE, and C). The bi-factor model is another form of hierarchical analysis, but unlike second-order model, the overarching dimension exerts direct effects only on the items and orthogonality between all of latent dimensions was imposed [35,53]. According to guidelines, comparisons were carried out by using the test differences in three fit indices, with the following criteria as cutoffs for model equivalence: ΔCFI (<0.010), and ΔRMSEA (<0.015 [35,54–57]). Overrunning this cutoff in two out of these three indices is evidence of model worsening.

In addition, considering that the PSIS-Y-SF is a new instrument, items were tested regarding their ability to discriminate subjects with low or high sport skills [58,59]. According to Ebel [58] and Chiorri [59] for typical performance test items (*e*.*g*., Likert scale), the maximum total score and quartile rank for each subject were calculated. Subsequently, a series of independent sample *t*-test–and their effect size (Cohen’s *d*) [60]–were computed to determinate item discriminating power by using as dependent variable the total score of the scale and the lowest and the highest quartile as grouping variable [58,59]. Item-total correlation (adjusted) was also computed.

Due to possible differences in the magnitude of factor loadings, Raykov’s MR [61] was chosen as measure of internal consistency of each single factor reliability–instead of both Cronbach’s *alpha* and Composite Reliability [61–64].

Multivariate Analysis of Variance (MANOVA) was performed to determine possible differences between players category (male youth *vs*. male *junior vs*. female youth *vs*. female *junior*–independent variable) simultaneously on the PSIS-Y-SF subscales (dependent variables). According to guidelines [65–67], both univariate and multivariate normality were assessed as well as the equality of covariances matrices and the absence of multicollinearity. Finally, the Games-Howell test was chosen for performing post-hoc analysis [65,66,68,69].

Finally, ordinary least square (OLS) regression analyses were performed to evaluate possible associations and differences between psychological skill and sport training variables: number of hours per week, number of years involved in volleyball, and years in the sport.

## Results

### Structural validity and psychometric properties

Item analysis revealed a non-perfect univariate normal distribution of some indicators (Table 1). Indeed, skewness ranged from -1.901 (item#13) to 0.846 (item#18) with a mean_sk_ of -0.033 and an SD_sk_ of 1.042; multivariate skewness (*b* = 47.281; *P*<0.001). Kurtosis ranged from -0.876 (item#10) to 3.261 (item#13) with a mean_k_ of -0.009 and an SD_k_ of 1.031; multivariate kurtosis (421.4391; *P*<0.001).

**Fig 1:**
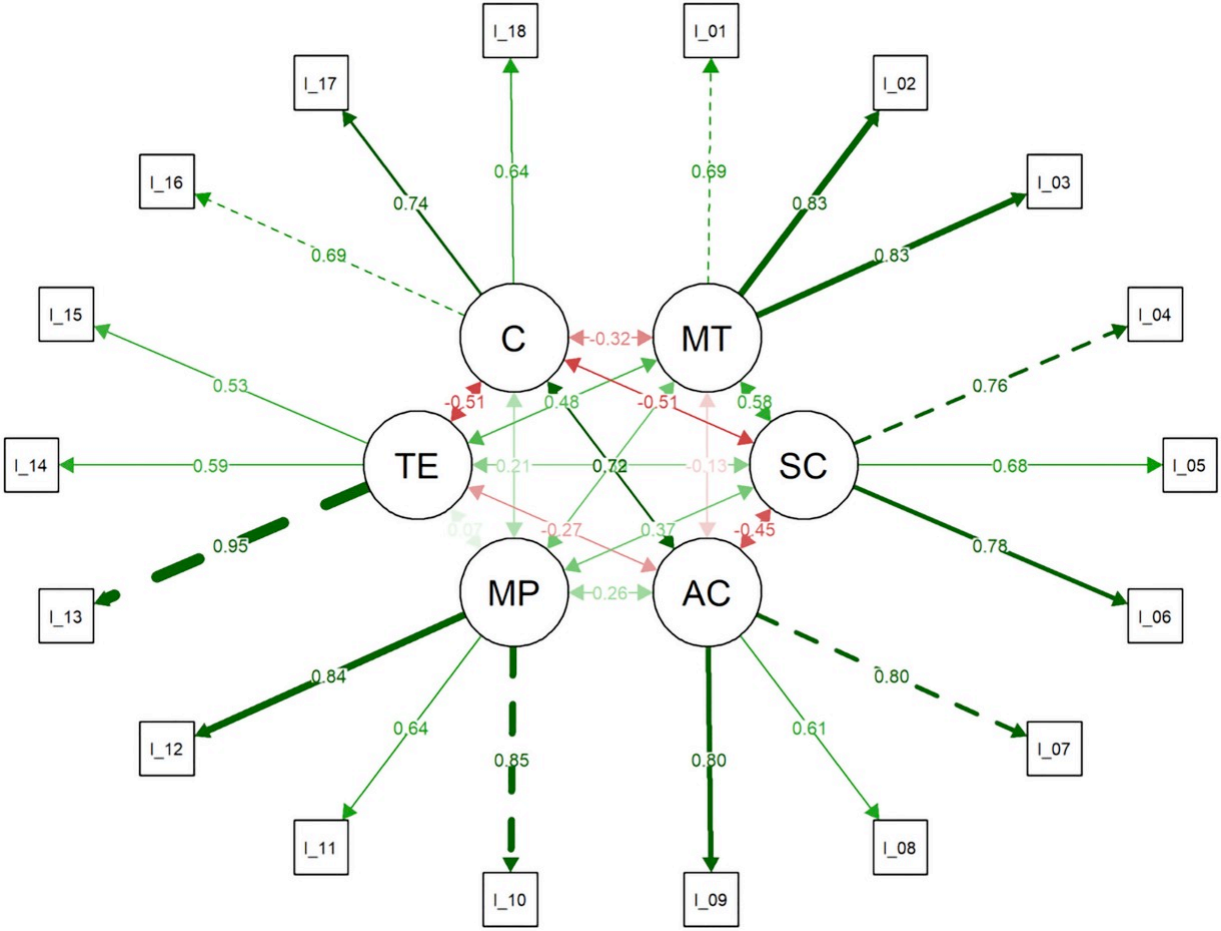
Graphical representation of the confirmatory factor analysis.

Results from the CFA suggest an adequate six first-order factor solution for the PSIS-Y-SF. Despite the χ^2^ was statistically significant [χ^2^(120) = 276.843; *P*<0.001], the others fit indices overcame the threshold for good model fit. Indeed, the RMSEA was equal to 0.066 [90%CI: 0.056–0.076; *P*(RMSEA<0.05) = 0.006], the CFI was equal to 0.974, and the WRMR was equal to 1.109. Moreover, each item loaded on the factor associated with itself: mean_loadings_ = 0.737; SD_loadings_ = 0.109 and ranging from 0.535 (item#15) to 0.953 (item#13). Consequently, items’ explained variance (*R*^2^) ranged between 0.286 (item#15) and 0.908 (item#13) with a mean_R2_ equal to 0.555 and an SD_R2_ equal to 0.161 (Table 2, Fig 1). Factor correlation matrix is displayed in S1 Table.

**Table 2:** Confirmatory Factor Analysis (CFA), item discriminant power (IDP), and Item-Total Correlation–adjusted (IT-TOT).

	CFA							IDP		IT-TOT
	MT(λ)	SC(λ)	AC(λ)	MP(λ)	TE(λ)	C(λ)	*R*^2^	*t*_i_	*d*	*r*_*Adj*_
Item1	0.692*	-	-	-	-	-	0.479*	-15.885*	2.629	0.477*
Item2	0.831*	-	-	-	-	-	0.691*	-24.091*	3.988	0.641*
Item3	0.835*	-	-	-	-	-	0.697*	-19.474*	3.223	0.631*
Item4	-	0.765*	-	-	-	-	0.585*	-21.111*	3.474	0.578*
Item5	-	0.683*	-	-	-	-	0.466*	-16.721*	2.744	0.547*
Item6	-	0.783*	-	-	-	-	0.614*	-19.436*	3.253	0.592*
Item7	-	-	0.795*	-	-	-	0.632*	-18.954*	3.170	0.564*
Item8	-	-	0.613*	-	-	-	0.375*	-16.429*	2.767	0.509*
Item9	-	-	0.799*	-	-	-	0.639*	-21.015*	3.507	0.616*
Item10	-	-	-	0.847*	-	-	0.717*	-28.919*	4.866	0.672*
Item11	-	-	-	0.642*	-	-	0.413*	-22.144*	3.715	0.531*
Item12	-	-	-	0.838*	-	-	0.703*	-20.825*	3.496	0.658*
Item13	-	-	-	-	0.953*	-	0.908*	-11.475*	1.814	0.455*
Item14	-	-	-	-	0.585*	-	0.343*	-20.605*	3.287	0.461*
Item15	-	-	-	-	0.535*	-	0.286*	-18.597*	2.966	0.394*
Item16	-	-	-	-	-	0.695*	0.482*	-15.204*	2.309	0.473*
Item17	-	-	-	-	-	0.740*	0.548*	-19.485*	2.949	0.524*
Item18	-	-	-	-	-	0.637*	0.406*	-15.179*	2.299	0.457*

* *P*<0.001; λ = factor loadings; *R*^2^ = explained variance; *t*_i_ = independent sample *t*-test; *d* = effect size; *r*_*Adj*_ *=* item-total correlation–adjusted.

The IDP analysis showed that each of the 18 items discriminated well between subjects with individuals with low and individuals with high sport psychological skills (Table 2). The discrimination parameter *t*_i_ ranged from -11.475 (item#13) to -28.919 (item#10), with an associated effect size (Cohen’s *d*) that ranged respectively from 1.814 (item#13) to 4.866 (item#10)–mean_*t*_ = -19.197; SD_*t*_ = 3.879; mean_*d*_ = 3.136; and SD_*d*_ = 0.695. Thus, all the discrimination values revealed a strong relation between each item and the corresponding sport psychological skill.

Then, considering these results, the six first-order factor solution factor solution was compared with different competing models that could better explain the PSIS-Y-SF factorial structure [35,53,55]. As reported in Table 3, model comparisons revealed the superiority of the hypothesized solution, i.e., six first-order factor model–accounting for six different dimensions. Consequently, this factorial solution was chosen to perform successive analysis.

**Table 3:** Model comparisons.

		χ^2^(*df*);	Comparison	RMSEA	|ΔRMSEA|	CFI	|ΔCFI|
1	Six-factor model	276.843 (120)	-	0.066	-	0.974	-
2	One-factor model	2360.686 (135)	1 *vs*. 2	0.233	0.168	0.636	0.339
3	Second order factor model	806.221 (129)	1 *vs*. 3	0.132	0.066	0.889	0.085
4	Bi-factor model	No Convergence	1 *vs*. 4	-	-	-	-

χ^2^ = Chi-square test; *df* = degree of freedoms; |Δ(…)| = absolute difference; RMSEA = Root mean square error of approximation; CFI = Comparative fit index.

As reported in Table 4, correlation analyses–between the six factors of the PSIS-Y-SF–were also performed. As reported in Table 4, statistically non-significant to moderate correlations between factors were found. Correlations ranged from 0.084 (correlation between TE and MP) to 0.497 (correlation between C and AC). Moreover, correlation analyses were performed separately across sex (male *vs*. female) and championship category (youth *vs*. *junior*). Results were showed in Table 4.

**Table 4:** Correlations between subscales of the Psychological Skills Inventory for Sports–Youth version–Short Form for the overall sample and divided by sex (males *vs*. females) and Championship Category (youth *vs*. *junior*).

		**Overall sample**					
		MT	SC	AC	MP	TE	C
1	MT	-					
2	SC	0.414***	-				
3	AC	-0.088	-0.326***	-			
4	MP	0.287***	0.272***	0.204*	-		
5	TE	0.309***	0.201***	-0.144*	0.084	-	
6	C	-0.221***	-0.346***	0.497***	0.171**	-0.309***	-
		**Male** (*under* the diagonal) *vs*. **Female** (*over* the diagonal)					
		MT	SC	AC	MP	TE	C
1	MT	-	0.449***	-0.075	0.383***	0.308***	-0.130
2	SC	0.424***	-	-0.283***	0.322***	0.293***	-0.390***
3	AC	-0.132	-0.356**	-	0.196**	-0.177*	0.473***
4	MP	0.176	0.16*	0.251	-	0.147*	0.144*
5	TE	0.299**	0.126	-0.130	0.010	-	-0.344***
6	C	-0.312***	-0.356***	0.586***	0.196*	-0.251**	-
		**Youth** (*under* the diagonal) *vs*. ***Junior*** (*over* the diagonal)					
		MT	SC	AC	MP	TE	C
1	MT	-	0.486***	-0.101	0.323***	0.348***	-0.197*
2	SC	0.339***	-	-0.396***	0.268***	0.218**	-0.325***
3	AC	-0.085	-0.240**	-	0.217**	-0.167*	0.511***
4	MP	0.269**	0.278**	0.216**	-	0.074	0.309***
5	TE	0.302***	0.181*	-0.086	0.051	-	-0.319***
6	C	-0.277**	-0.380***	0.468***	0.034	-0.252**	-

Note: Motivation (MT), Self-Confidence (SC), Anxiety Control (AC), Mental Preparation (MP), Team Emphasis (TE), and Concentration (C)

* *P*<0.050

** *P*<0.010

*** *P*<0.001.

Finally, the MR suggested a good internal consistency for all the scales: MT = 0.845; SC = 0.794; AC = 0.803; MP = 0.848; TE = 0.915; and C = 0.739.

#### Multivariate Analysis of Variance

As reported in Table 1, skewness ranged from -0.851 (TE) to 0.485 (C) and kurtosis ranged from -0.629 (MP) to 0.550 (TE). According to guidelines [65–67], Mahalanobis distance was performed for each subject and compared with the critical χ^2^ value of 22.46 (*df* = 6, derived from the number of dependent variables; α = 0.001). This comparison revealed the presence of a single multivariate outlier and five multivariate influential cases (2.0% of the total sample) with Mardia’s Kurtosis equal to 5.078; *P*<0.001. However, considering this negligible percentage these subjects were retained into the analyses.

Correlations between dependent variables were under the threshold for collinearity (0.8), as showed in Table 4. Finally, the Box’s M test was equal to 86.594 (*F* = 1.321; *P* = 0.045) revealing that the covariance matrices were not homogeneous. However, according to guidelines, MANOVA is quite robust to small violations of assumptions [65–67]. Consequently, MANOVA revealed a statistically significant difference between players category (male youth *vs*. male *junior vs*. female youth *vs*. female *junior*) on the PSIS-Y-SF subscales: Wilks’s λ = 0.734, *F* = 5.126, and *P*<0.001, η_p_^2^ = 0.094 –suggesting the effect of gender and age on the sport psychological skills. MANOVA was performed also removing influential cases. Mardia’s Kurtosis was equal 1.538; *P*>0.050 ns; Box’s M test = 88.446 (*F* = 1.348; *P* = 0.034); Wilks’s λ = 0.726, *F* = 5.436, and *P*<0.001, η_p_^2^ = 0.101. Games-Howell post hoc analysis showed that statically significant univariate contrasts in the previous analysis (with the overall sample) were retained even though influential cases were removed. In order to test differences between player category groups within PSIS-Y-SF subscales, analyses of variance (ANOVA) with focused contrasts were conducted for each dependent variable–as shown in Table 5.

**Table 5:** Mean (standard deviation, SD) for the Psychological Skills Inventory for Sports–Youth version–Short Form subscales, results of Multivariate Analysis of Variance.

	MY–Male Youth	MJ–Male *Junior*	FY–Female Youth	FJ–Female *Junior*			
	Mean (SD)	Mean (SD)	Mean (SD)	Mean (SD)	*F*	η_p_^2^	Games-Howell’s post-hoc contrasts
MT	4.261(0.804)	3.821(0.924)	4.082(0.754)	4.235(0.698)	4.272**	0.041	FJ>MJ*; MY>MJ*
SC	3.810(0.731)	3.385(0.787)	3.182(0.809)	3.415(0.706)	7.773***	0.072	MY>FY***; MY>FJ*; MY>MJ*
AC	2.582(0.933)	2.554(0.932)	2.884(0.925)	2.691(0.871)	2.256	0.022	-
MP	2.830(0.878)	2.913(0.974)	2.594(1.034)	2.890(0.959)	2.072	0.020	-
TE	3.967(0.897)	3.985(0.785)	3.909(0.748)	4.378(0.536)	7.124***	0.067	MY<FJ**; MJ<FJ*; FY<FJ***
C	2.457(0.943)	2.364(0.799)	2.355(0.831)	2.012(0.616)	4.534**	0.043	FY>FJ**; MY>FJ* MJ>FJ*

*** *P*<0.001

** *P*<0.010

* *P*<0.050; η_p_^2^ = effect size.

Note: AC and C are reversed score scales: higher values in the subscale corresponded to lower anxiety control (AC) and concentration (C), respectively.

More in detail, no overall statistical differences emerged between the specific player categories in the Anxiety Control subscale (*F* = 2.256, *P* = 0.082 *ns*; and η_p_^2^ = 0.022) as well as in the Mental Preparation subscale (*F* = 2.072, *P* = 0.104 *ns*; and η_p_^2^ = 0.020).

Moreover, mean differences were found within the other subscales. Indeed, the “male *junior*” group (mean = 3.821, SD = 0.924) showed statistically significant lower values in the Motivation subscale than the “male youth” group (mean = 4.261, SD = 0.804), and the “female youth” and the “female *junior*” group (mean = 4.235, SD = 0.754): *F* = 4.272, *P* = 0.006; and η_p_^2^ = 0.041.

Moreover, the “male youth” group (mean = 3.810, SD = 0.731) showed statistically significant higher values in the Self-Confidence subscale than the “male *junior*” group (mean = 3.385, SD = 0.787), the “female youth” group (mean = 3.182, SD = 0.809), and the “female *junior*” group (mean = 3.415, SD = 0.706): *F* = 7.773, *P*<0.001; and η_p_^2^ = 0.072.

In addition, the “female *junior*” group (mean = 4.378, SD = 0.536) showed statistically significant higher values of the Team Emphasis subscale than the “male youth” group (mean = 3.967, SD = 0.897), than the “male *junior*” group (mean = 3.985, SD = 0.785), and the “female youth” group (mean = 3.909, SD = 0.748): *F* = 7.124, *P*<0.001; and η_p_^2^ = 0.067.

Finally, both the “female *junior*” group (mean = 2.012, SD = 0.616) showed statistically significant lower values of the Concentration subscale than the “male youth” group (mean = 2.457, SD = 0.943), the “male *junior*” group (mean = 2.364, SD = 0.799), and the “female youth” group (mean = 2.355, SD = 0.318): *F* = 4.534, *P* = 0.004; and η_p_^2^ = 0.043.

#### Associations between sport psychological skills and training variables

As reported in Table 6, OLS regressions revealed an association between years of training and the Team Emphasis subscale (*B* = 0.215; *t* = 3.817; *P*<0.001) as well as the Concentration subscale (reverse scored scale; *B* = -0.144; *t* = -2.535; *P* = 0.012). No statistically significant associations between the others sport psychological skills and years of training were found (Table 6).

**Table 6:** Standardized Regression coefficients, *t*-tests and *P*-values for univariate OLS regression analyses for the Psychological Skills Inventory for Sports–Youth version–Short Form subscales and sport training variables.

	Hours of training per week			Years of training		
	Standardized *β*	*t*-test	*P*-value	Standardized *β*	*t*-test	*P*-value
MT	0.274	4.945	*P*<0.001	0.051	0.893	*P* = 0.372 *ns*
SC	0.275	4.964	*P*<0.001	0.070	1.221	*P* = 0.223 *ns*
AC	-0.079	-1.380	*P* = 0.169 *ns*	-0.058	-1.011	*P* = 0.313 *ns*
MP	0.211	3.757	*P*<0.001	0.080	1.397	*P* = 0.163 *ns*
TE	0.135	2.373	*P* = 0.018	0.215	3.817	*P*<0.001
C	-0.121	-2.123	*P* = 0.035	-0.144	-2.535	*P* = 0.012

Note: AC and C are reversed score scales: higher valuesin the subscale corresponded to low anxiety control (AC) and concentration (C), respectively.

In addition, OLS regressions revealed no association between Anxiety Control subscale and the number of hours of training (*B* = -0.079; *t* = -1.380; *P* = 0.169). Opposite, OLS regressions revealed a negative association between the Concentration subscale (reverse scored scale) and the number of hours of training per week (*B* = -0.121; *t* = -2.123; *P* = 0.035) suggesting that an improvement training hours, could improve concentration’s skills. At the same time, there were positive associations between the number of hours of training per week and motivation (*B* = -0.274 *t* = 4.945; *P*<0.001), Self-Confidence (*B* = 0.275; *t* = 4.964; *P*<0.001), Mental Preparation (*B* = 0.211; *t* = 3.757; *P*<0.001) and Team Emphasis (*B* = 0.134; *t* = 2.373; *P* = 0.018). These results suggested that an increase in training hours was associated to sport higher sport psychological skills.

All relevant data are within S1 Dataset.

## Discussion

In sports practice, top athletic achievements or unexpectedly poor achievements are often attributed to athletes’ psychological skills. In line with the Achievement Goal Theory [1,2] and Self-Determination Theory [3], in last years, physical as well as psychological research highlighted the key role of psychological sport skills to endorse better athletes’ performance [3–5,70]. This has led researchers to focus more closely on the relationship between the athlete's psychological abilities and their performance–showing how success depends on both physical and technical components and psychological factors.

Within this context, the goal of this study was the development and initial validation of a short form of the PSIS-Y (PSIS-Y-SF): a new tool for measuring–in a more concise and precise way–psychological skills in athletes [34].

CFA successfully confirmed the six first-order factors of the original PSIS-Y–Motivation, Self-Confidence, Anxiety Control, Mental Preparation, Team Emphasis, and Concentration–even in this short form. Also, the CFA suggested that the PSIS-Y-SF had good fit indices and a good structural validity, despite the WRMR was slightly over the recommended cut-off. Furthermore, items’ factor loading revealed a strong relation between the items and the corresponding latent sport psychological skill [35,52–54].

Moreover, this factorial structure was compared with several competing models–a single factor model, a second factor model, and a bi-factor one [35,53–57]–and even in this case, results suggested the higher adequacy of the hypothesized six first-order structure.

In addition, the item discrimination power was assessed. Results of this analyses highlighted that each of the 18 items composing the PSIS-Y-SF well discriminated between subjects with low and individuals with high sport psychological skills–suggesting the goodness of the item to capture individuals with different levels of the skill as well as the ability of each single item to represent its underlying construct.

Reliability analysis was also performed providing satisfying results. Considering that the PSIS-Y-SF is a new tool assessing sport psychological skills, Cronbach’s *alpha* was considered not adequate–due to the unknown possible (strong) differences in items’ factor loading [64]. Thus, a more trustworthy weighted method to estimates reliability (MR [61–63]) was used: this index suggested a moderate to high internal consistency–perfectly in line with the previous (long) versions of the inventory [31]. Correlations between subscales were small to moderate, suggesting a different degree–and a different strength–of association between sport psychological skills.

In Table 5, mean scores and standard deviations are shown for each between-player category (male youth *vs*. male *junior vs*. female youth *vs*. female *junior*) in relation to each sport psychological skill. The analysis of multivariate variance and the Games-Howell post hoc contrasts analyses of variance revealed statistical differences between the four aforementioned player categories for all of the PSIS-Y-SF subscales–with the exception of the AC and MP subscales. These results are in line with the original PSIS version that showed good discriminant ability [8]. Moreover, these results are coherent with previous studies that suggest gender and age differences in motivation [71,72] as well as self-confidence, concentration, team emphasis, and mental preparation [7,8,72]. More in detail, considering the focused contrasts, in the Motivation subscale the “male *junior*” group showed a lower motivation than all of the other players’ categories (“female *junior*”, “female youth”, and the “male youth” group). Regarding the Self-Confidence subscale, the “male youth” group showed statistically significant higher values than the “male *junior*” group–as described by previous studies [8]. At the same time, in the Team Emphasis subscale, the “female *junior*” group showed statistically significant higher values than all of the others groups. Finally, regarding the Concentration subscale (reversed score), “female *junior*” group showed a better ability to concentrate than all of the other players categories (“female youth”, “male youth” and the “male *junior*” group).

Also, OLS regression analyses provided interesting findings. Indeed, on one hand, they revealed a statistically non-significant linear association between the majority of sport psychological skills and years of training. On the other hand, in line with literature, OLS regressions pointed out that a higher amount of training hours is associated with higher sport psychological skills (Table 6).

Despite these promising findings, several limitations have to be highlighted to this study. First of all, despite the sample size was adequate to correctly perform a CFA, it was too small to perform measurement invariance analyses. However, it should be highlight that measurement invariance analysis was not the main purpose of this study–indeed, this research aimed to develop a short form of a scale and to test its psychometric proprieties. Moreover, the “small sample size issue” could be solved by future researches that should increase the sample size. Moreover, the lack of a second measurement over the time does not allow performing longitudinal analyses as test-retest reliability, temporal stability, and longitudinal invariance. Future studies could address this issue by creating longitudinal research designs. Such designs are necessary anytime research aim is determining which psychological factors are important for successful sport performance over athlete’s development from beginner to elite. As well as, longitudinal research designs should be preferred when determining which youth athlete’s psychological factors could predict successful sport performance in elite athlete. In addition, it has to be underlined that this research was based on a sample composed by volleyball-players only and that no other questionnaires were administered. These two issues limit the generalizability (external validity) of the findings discussed above. Thus, future research studies should resolve this issue by administering a complete tests battery to a more heterogeneous sample. Moreover, a classical *P*-value approach was used in this study (*P*<0.050). However, according to the current debate on the usage of statistical significance [73], further indices (*e*.*g*., effect sizes) and/or statistics (*e*.*g*., uncertainty intervals) should be taken into account in order to carefully evaluate (*non-*)significant result as evidence for (no) effect/association. Finally, despite in the scale construction process items’ semantic redundancy was strongly avoided, the use of only three items per subscale may have limited the measurement of the construct in question–which should therefore have been defined too narrowly. Moreover, another issue should be taken into consideration regarding brief scales. Despite–in this case–reliability indices were higher than acceptable values; brief scales could hide reliability problems. Indeed, a small number of items could lead to obtain reliability coefficients below necessary standards.

However, despite these limitations, the preliminary validation of the PSIS-Y-SF indicates that it could be a useful instrument in measuring an individual’s sport psychological skills. Indeed, given the shortness of this instrument, it is likely to have high utility in both psychological and sport settings for the assessment of sport psychological skills as a core feature of performance as well as monitoring progress over the time. Moreover, the PSIS-Y-SF could be added in the planning of psychological intervention [55]. Indeed, on one hand, it could be used for the reduction of fatigue [74–78] and/or to improve the motivation to change [79–82] and/or to assess the need of social support to reduce psychological distress [83–86]. On the other hand, it could be used to increase athletes’ self-esteem [87–90] and self-awareness [91–93] as well as emotions, flourishing, and well-being [94–98].

Moreover, this inventory provides a starting point for planning sport psychological intervention, because it provides an opportunity for sport psychologists–and other sport professional figures–to use the lower scored factors to convey to the athlete their understanding of these issues and a willingness to work with them on these difficulties.

Besides this utility, the PSIS-Y-SF provides a valid and reliable instrument to be used in research in providing six measures of performance predictors which can be tracked for changes over the time, especially following (psychological) treatment.

## Conclusions

The present research has a possibility of practical application of its findings in the measurement of psychological skills of groups of players in volleyball clubs and national volleyball associations. By means of this measure, coaches in clubs and national associations could get support in making decisions about orientation of individual players for performing certain roles or player specialization at *junior* age.

It could be concluded that there is an initial strong indication supporting the use of the PSIS-Y-SF, with additional item selection (i.e., item analysis), as a quality-measuring instrument for measuring psychological skills in sports (i.e., volleyball players). Thus, this preliminary validation of the PSIS-Y-SF indicated that this instrument could be considered as a useful tool to measure individual’s psychological skill in sports.

## Supporting information

S1 Dataset All relevant data. (XLSX)

S1 Table Factor correlations matrix. (DOCX)
