# Supplementary material for: Development and factorial validity of the Psychological Skills Inventory for Sports, Youth Version – Short Form: Assessment of the psychometric properties
Source: PLoS One. 2019 Aug 15;14(8):e0220930. doi: 10.1371/journal.pone.0220930 (PMC6695110; doi:10.1371/journal.pone.0220930)
Supplement: S1 Table — (DOCX) [file pone.0220930.s002.docx]

**S1 Table. Factor correlations matrix.**

|  |  | **Overall sample** | | | | | |
| --- | --- | --- | --- | --- | --- | --- | --- |
|  |  | MT | SC | AC | MP | TE | C |
| 1 | MT | - |  |  |  |  |  |
| 2 | SC | 0.575*** | - |  |  |  |  |
| 3 | AC | -0.130*** | -0.454*** | - |  |  |  |
| 4 | MP | 0.388*** | 0.368*** | 0.255*** | - |  |  |
| 5 | TE | 0.480*** | 0.314*** | -0.273*** | 0.069 | - |  |
| 6 | C | -0.324*** | -0.506*** | 0.723*** | 0.211 | -0.510*** | - |

Note: Motivation (MT), Self-Confidence (SC), Anxiety Control (AC), Mental Preparation (MP), Team Emphasis (TE), and Concentration (C); * *P*<0.050; ** *P*<0.010; *** *P*<0.001.
